# Co-option of Hair Follicle Keratins into Amelogenesis Is Associated with the Evolution of Prismatic Enamel: A Hypothesis

**DOI:** 10.3389/fphys.2017.00823

**Published:** 2017-10-24

**Authors:** Elia Beniash

**Affiliations:** Department of Oral Biology, University of Pittsburgh, Pittsburgh, PA, United States

**Keywords:** enamel, keratin 75, evolution, enamel rod, hair follicle

## Abstract

Recent discovery of hair follicle keratin 75 (KRT75) in enamel raises questions about the function of this protein in enamel and the mechanisms of its secretion. It is also not clear how this protein with a very specific and narrow expression pattern, limited to the inner root sheath of the hair follicle, became associated with enamel. We propose a hypothesis that KRT75 was co-opted by ameloblasts during the evolution of Tomes' process and the prismatic enamel in synapsids.

Since early days of enamel research the question regarding the presence of keratins in this epithelial tissue intrigued scientists (see Duverger et al., 2016 for review). A number of studies suggested that keratins are present in the insoluble and heavily cross-linked matrix of mature enamel, however due to the extreme insolubility of this material these studies were not able to identify these keratins (Robinson et al., 1975, 1989a,b; Robinson and Hudson, 2011). Recently, Keratin 75 (KRT75) was identified in ameloblasts and the mature enamel matrix (Duverger et al., 2014). Importantly, it was found that a single amino acid substitution in this protein, which causes a hair condition pseudofoliculitis barbae, or barber rush, affects structural and mechanical properties of enamel and increases caries susceptibility (Duverger et al., 2014), suggesting an important functional role for Krt75 in amelogenesis. At the same time a number of critical questions regarding Krt75 need to be investigated. The fact that Krt75 is a cytosolic protein, lacking the signaling peptide, essential for proper sorting of secretory proteins, raises the fundamental question regarding the mechanism of its secretion. One possible scenario is that cytosolic proteins end up in enamel with the vestiges of the Tomes' processes (Warshawsky and Vugman, 1977). Another question is- what role of this highly specialized protein, expressed almost exclusively in the inner root sheath and companion layer of the hair follicle (Winter et al., 1998), plays in enamel? The later question is especially interesting from the evolutionary perspective, since primitive enamel appeared prior to the sea-land transition and the evolutionary explosion of keratins in basal tetrapods.

It is a widely accepted that, ectodermal appendages, such as teeth and hairs evolved independently, but share a common developmental blueprint (Sharpe, 2001). Specifically, the role of epithelial-mesenchymal interactions is absolutely critical for the development of these organs, and their patterning and morphogenesis involve a number of shared regulatory pathways (Biggs and Mikkola, 2014; Lan et al., 2014). These pathways are evolutionary conserved and are involved in morphogenesis of other ectodermal appendages, such as elasmobranch teeth (Rasch et al., 2016) or teleost scales (Sharpe, 2001), which are not direct evolutionary homologs of mammalian teeth or hairs (Qu et al., 2015; Braasch et al., 2016). Although these disparate organs utilize common morphogenetic blueprint, the structural proteins of these appendages differ significantly and in many instances have evolved independently. The presence of Krt75 in the mammalian teeth represents an evolutionary puzzle. It is established that the teeth covered with true enamel appeared in the common ancestors of sarcopterygians prior to the sea to land transition and that the true enamel present in all classes of tetrapods (Qu et al., 2015; Braasch et al., 2016), while the evolutionary explosion of keratins occurred in basal tetrapods in connection with the sea to land transition and adaptations to multiple land habitats (Vandebergh and Bossuyt, 2012). Intriguingly, in Anura close orthologs of hair and hair follicle keratins are expressed in toe pads and claws, suggesting that the expansion of these genes is associated with the evolution of ectodermal appendages in crown tetrapods (Vandebergh et al., 2013). Although KRT75 gene was not found in amphibians, it is present in all extant amniotes. In birds Krt75 is found in the cells of the feather follicle but not in the feathers themselves, which are made mainly of beta-keratins, a specialized family of proteins found in reptiles and birds (Ng et al., 2012; Greenwold et al., 2014). A mutation of Krt75 in chicken leads to defects in feather rachis, causing so-called frizzle feather phenotype (Ng et al., 2012). These observations draw some interesting commonalities between Krt75 in mammals and birds, namely their localization in the follicles but not in hairs and nails themselves and their control of hair and feather morphology (Ng et al., 2012; Jasterzbski and Schwartz, 2015). This gene also exist in lizards however its tissue localization is unknown (Eckhart et al., 2008).

KRT75 is present in a wide variety of mammals, which is not surprising since it plays a major role in hair formation. Whales (Cetacea), which are hairless, lost a number of hair and hair follicle keratin genes (Nery et al., 2014). Interestingly, a recent study of keratin genes in 6 mammalian species with annotated genomes showed that bottleneck dolphins (which lack hair but retain teeth) retained functional KRT75 gene, while in the toothless and hairless minke whales, this gene is silent (Nery et al., 2014). Similarly, in pangolins which are toothless animals, covered in scales, KRT75 is functional, however there are two single amino acid substitutions in a highly conserved region of the protein (Choo et al., 2016). These findings suggest that KRT75 is important for tooth formation. However, what is the potential role of (Biggs and Mikkola, 2014) this protein in the mammalian teeth? This question remains unclear. There are several major differences in tooth morphology and ultrastructure between mammals and other toothed tetrapods. Among them is the presence of thick prismatic enamel, with a sophisticated decussating pattern, while other extant tetrapods present with prismless enamel (Sander, 2000). According to Sander, development of prismatic enamel occurred after the separation of synapsids from other branches of amniotes (Sander, 1997). Enamel rod, the basic building blocks of the prismatic enamel, is a secretory product of Tomes' process, a highly specialized cellular secretory apparatus (Sander, 1997). Cross-sectional profiles and shapes of the enamel rods are determined by the organization of Tomes' processes and trajectories of ameloblasts movements during the appositional grows of secretory enamel. Importantly, ameloblasts equipped with Tomes' processes are only present in mammals and are not found in other extant toothed tetrapods (Sander, 2000). The facts presented above support a hypothesis that Krt75, and potentially other hair follicle keratins, were co-opted by ameloblasts during the evolution of Tomes' process and the prismatic enamel, which is the major evolutionary innovation (Figure 1). The observation that a single amino acid substitution in Krt75 causes malformation of the enamel rods (Duverger et al., 2014) further supports this notion. It has to be pointed out that, as of now, we do not have enough information regarding the exact function of Krt75 in enamel and the evolutionary modifications of the mammalian KRT75 to draw any conclusions. The goal of this essay was to provoke interest in the research community to this intriguing possibility of co-option of a highly specialized hair follicle keratin into enamel.

**Figure 1:**
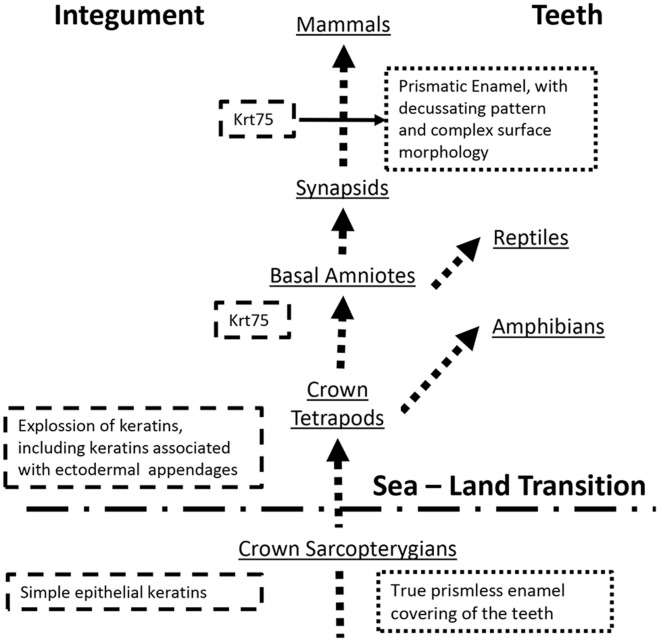
A Schematic diagram illustrating the proposed hypothesis.

## Author contributions

The author confirms being the sole contributor of this work and approved it for publication.

### Conflict of interest statement

The author declares that the research was conducted in the absence of any commercial or financial relationships that could be construed as a potential conflict of interest. The reviewer CR and handling Editor declared their shared affiliation.
